# Patient and clinician perspectives on digital tools in heart failure: results in three London hospital networks (Qual-HF)

**DOI:** 10.1093/ehjdh/ztag146

**Published:** 2026-09-14

**Authors:** Kyle McBeath, Jillian P Riley, Martin R Cowie

**Affiliations:** Cardiology Department, Royal Brompton Hospital, Sydney Street, London SW3 6NP, UK; King’s College London, Strand, London WC2R 2LS, UK; Imperial College London, South Kensington Campus, London SW7 2AZ, UK; King’s College London, Strand, London WC2R 2LS, UK; Imperial College London, South Kensington Campus, London SW7 2AZ, UK

**Keywords:** Heart failure, Digital health, Qualitative research, Digital literacy, Technology adoption, Self-management

## Abstract

**Aims:**

There are limited data on the experience and perspectives of patients or clinicians about the use of digital tools (DTs, such as internet resources, social media, and smartphone apps) in heart failure (HF). This study aimed to explore patient and clinician perspectives of DT use and provide insights into the factors that facilitate or obstruct such use.

**Methods and results:**

This was a mixed-methods study using semi-structured interviews with 14 people living with HF and 9 clinicians working as HF specialists. Recorded interviews underwent thematic analysis. Three main themes emerged: ‘the dual nature of information’, the ‘recommendation gap’, and ‘self-care as a time-dependent behaviour’. Patients were generally fearful of information from DTs, with many disengaging from their use as a form of self-preservation. Patients described finding the ‘right’ information as a struggle, and their behaviour appeared related to their level of digital health literacy and degree of activation. Recommendation of DTs by clinicians was influenced by assumptions around the digital the capability of patients, the lack of evidence-based guideline recommendations, and a lack of local or international consensus around the use of DTs.

**Conclusion:**

Many patients living with HF are anxious about online information and withdraw from using DTs. A clinician’s recommendation may help, providing an ‘information prescription’, but is limited by assumptions about patients and lack of training, local consensus, and clear international guidance. Routine assessment of digital capability, particularly in older patients, may balance some of these assumptions and support more consistent and equitable use.

## Graphical Abstract

**Graphical Abstract:**
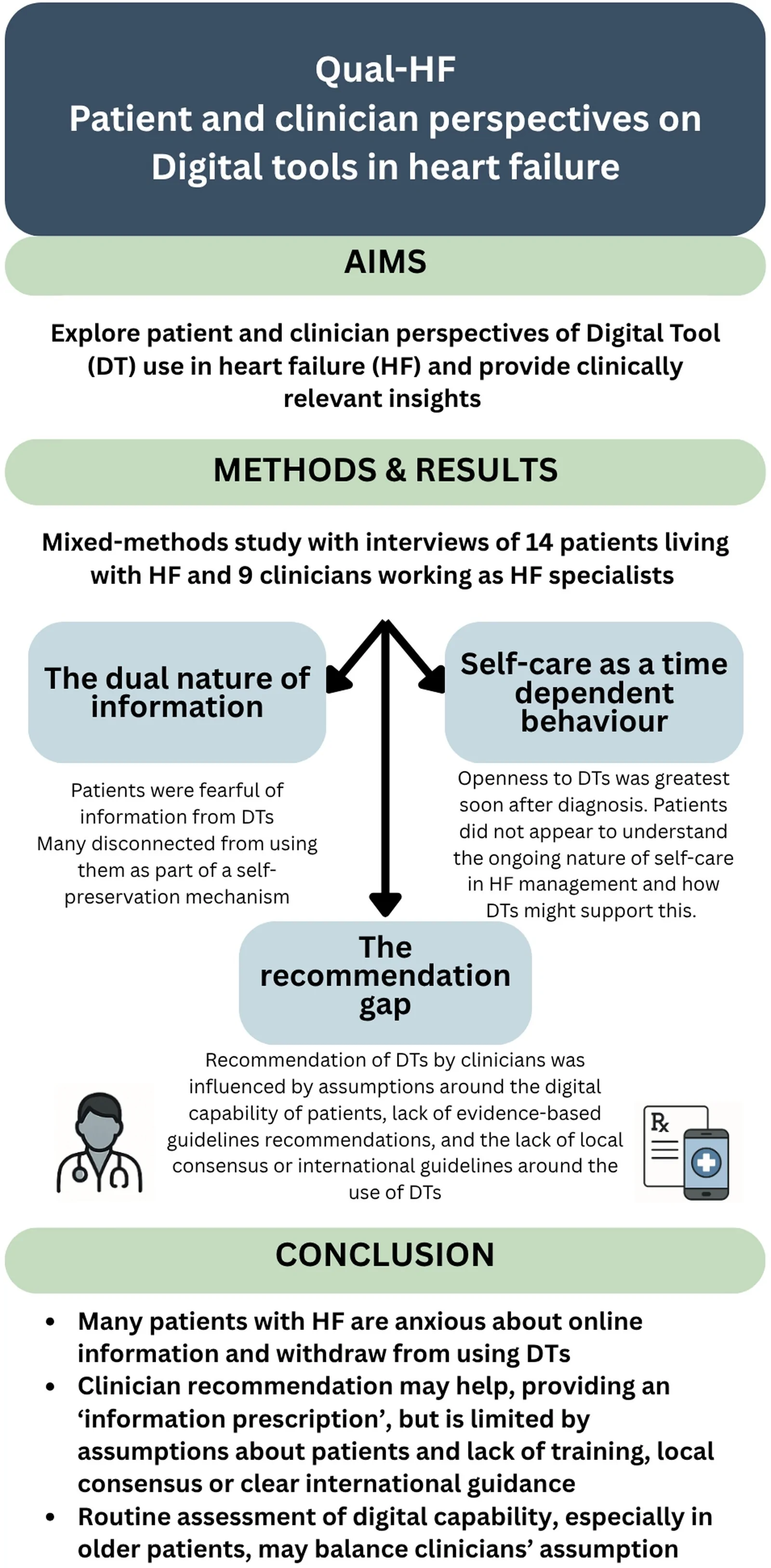

## Introduction

Digital technologies, such as digital tools (DTs; mobile apps, internet resources, and social media), can improve disease specific knowledge, support self-care, improve access to care, and improve the impact of interactions with healthcare professionals.^1^ As such, DTs have the potential to improve the overall illness experience for patients living with heart failure (HF).

European Society of Cardiology guidelines for Heart Failure recognize the potential importance of DT in successful disease management programmes,^2^ and recent Heart Failure Association (HFA) practical recommendations emphasize the need for personalized education and ongoing self-care support.^3^ The HFA developed and maintains the patient-friendly website, ‘Heartfailurematters.org’.

Clinician recommendation (or prescription) of DTs is likely important: it increases both the use and trust in these tools and helps ensure that appropriate DTs are used.^1^ Effective DT use is also shaped by patient factors, including digital health literacy—the ability to find, understand, evaluate, and apply online health information^4^—and patient activation: the knowledge, skills, and confidence to manage one's own health.

We aimed to explore patient and clinician perspectives of DT use in HF and gain insights into the facilitators and barriers that exist, particularly around the recommendation of DTs, an important factor highlighted in a previous quantitative study.^5^

## Methods

This was a mixed-methods study: quantitative data from the parent Digital-HF cohort^5^ informed both purposive sampling and interpretation of the qualitative findings.

The study population consisted of patients with HF and specialist HF clinicians who had attended or worked at three large teaching hospitals in London, UK. Those under the age of 18 or those who lacked proficiency in English were excluded.

Patient participants were selected from the Digital-HF cohort to represent a range of levels of activation, DT use, and digital health literacy (HL) .

Heart failure clinicians from the three hospitals were selected to represent a range of clinical experience and professional roles.

### Data collection

Semi-structured interviews were conducted via video conferencing platforms, Zoom™ and Microsoft Teams™, between January and March 2023. An interview schedule, informed by the findings of the parent Digital-HF study and by the study aims, guided each interview (mean duration 26 min, range 23–32). Interviews were conducted by the primary author (KM), an HF clinician with prior qualitative interview experience and no prior caring relationship with the participants. Interviews were recorded and automatically transcribed using digital software.

Sociodemographic and clinical characteristics, digital HL, and patient activation were collected from a previous study in which all participants had taken part.^5^

### Data analysis

Interviews were designed to gather ‘rich’ data using methods adapted from a constructivist grounded theory method, chosen to allow themes to be co-constructed between sequentially analysed patient and clinician accounts.^6^

Data analysis was performed according to the phases of thematic analysis.^7^ The first four interviews were co-coded (KM and JR), and a coding framework was developed. Interviews with patients and clinicians were performed sequentially, allowing themes to evolve with an asynchronous ‘dialogue’ between patient and clinician narratives. KM coded the remaining interviews, with the wider research group reviewing coding and theme development through regular discussion.

The study was approved by the Health Research Authority and Health and Care Research Wales (IRAS reference number: 307636). Written informed consent was obtained.

## Results

We approached 23 patients, 14 (61%) of those agreed to be interviewed. The patients had a median age of 64 (Interquartile range [IQR] 52–73) years, four (29%) were female, and five (36%) were non-white. Of these 14 patients, 11 (79%) used DTs for health. Six (43%) patients had low digital HL. Baseline characteristics and focused questionnaire data for all patient participants are summarised in Table 1.

**Table 1:** Baseline characteristics and focused questionnaire data for all patient participants in Qual-HF

Patient	Age (yr)	Sex	Ethnicity	Education	Length of HF (months)	Smartphone Owner	Recommended a DT	DT user	eHEALS (out of 40)	Low DHL	PAM (out of 100)
1	73	M	White British	Secondary school	74	Yes	No	No	39	No	77.7
2	51	F	White British	Degree	110	Yes	Yes	Yes	40	No	90.7
3	70	F	White British	Vocational qualification	13	Yes	Yes	Yes	31	No	58.1
4	43	M	White British	Vocational qualification	73	Yes	No	Yes	31	No	70.2
5	60	M	Mixed Ethnicity	Higher degree	98	Yes	No	Yes	32	No	60.6
6	81	M	White British	Vocational qualification	175	Yes	No	Yes	28	No	51
7	76	M	White British	Vocational qualification	204	Yes	No	Yes	22	Yes	43.7
8	65	F	Indian	Degree	37	Yes	No	No	20	Yes	38.1
9	52	F	Black other	Degree	24	Yes	Yes	Yes	30	No	55.6
10	63	M	Caribbean	Degree	6	Yes	No	Yes	24	Yes	63.1
11	49	M	White British	Vocational qualification	80	Yes	Yes	Yes	25	Yes	45.3
12	62	M	White British	Secondary school	145	Yes	Yes	Yes	20	Yes	53.2
13	80	M	White British	Vocational qualification	70	Yes	No	Yes	30	No	42.2
14	71	M	Caribbean	Secondary school	48	Yes	No	No	15	No	51
**Summary**	**64 (IQR 52–74)**	**28.6% female**	**35.7% non-white**	**36% University Education**	**73.5 (IQR 34–119)**	**100% ownership**	**35.7% recommended a DT**	**78.5% DT user**	**27.6** **±** **6.9**	**42.9% had low DHL**	**54.4 (IQR 38–62)**

HF, heart failure; DT, digital tool; eHEALS, eHealth Literacy Scale; DHL, digital health literacy; PAM, Patient Activation Measures; IQR, interquartile range.

Thirteen clinicians were approached via email. In total, nine (69%) of those approached were interviewed. Clinicians had a mean of 17.4 ± 8.8 years of clinical experience and included: four HF specialist nurses, four cardiology doctors (two in training), and one HF specialist pharmacist.

### Thematic analysis

All 23 patient and clinician interviews were merged for analysis. Three main themes emerged from the data.

#### The dual nature of information

The majority, 13 (93%), of patients perceived information from DTs as a risk, using words such as ‘*fear*’, ‘*anxiety’*, and ‘*danger’*. William (Patient 12), a 49-year-old man with low digital health literacy and very low activation, described how, once information has been seen, ‘*You can't unread it*’. He described this as activating a ‘self-preservation’ mechanism: disengagement from online HF information. This theme was persistent irrespective of age, sex, ethnicity, or measured literacy, but responses differed. Where patients had higher digital HL scores and activation, they found ways of managing DT use, such as returning to information when in a better frame of mind, narrowing the selection of DTs used, or using familiar sources. Edward (Patient 4) described that, ‘*At first I found all the bad things that can happen; it took me about a year to realize the positive things also*’.

Clinicians similarly viewed a diagnosis of HF and the wealth of online information available, as ‘*overwhelming’* to patients.

#### The recommendation gap

Five (56%) of the clinicians frequently recommended DTs, while nine (64%) patients described how they did not recall having been recommended to use any specific DT.

Patients assumed that if they had not been recommended a DT, clinicians had consciously decided that they were not suitable for DTs.

Seven of the clinicians (78%) interviewed described how they made assumptions about the patients’ digital literacy, capability, or desire to engage with DTs.

Five clinicians (56%) expressed the opinion that one of the main reasons for not recommending DT use was that it was not part of their standard consultation structure.

Clinicians recommending DTs did not describe any published literature around DTs and expressed concern that there was no clear guidance from national or international associations.

Of the nine clinicians, eight (89%) said that they would be more likely to recommend DTs if there was a local or departmental consensus on which DTs to recommend.

Clinicians downloaded and used DTs with no evidence for their own personal use, but did not recommend DTs for patients due to the same lack of evidence. No clinician mentioned ‘heartfailurematters.org’, despite being aware of the ESC heart failure guidelines.

#### Self-care as a time-dependent behaviour

Thirteen (93%) patients actively collected their own data via a smartwatch, smartphone, or other device. Seven (50%) used apps for self-monitoring.

Patients described how their openness to use DTs was dependent on where they were in the course of their illness. Those who were newly diagnosed were open to trying DTs but, as time went on, and patients either became symptomatically better or became more set in a routine of self-care behaviours, they were less inclined to try DTs.

There was limited understanding or acceptance that self-care is a constant process. Either through denial of their condition or because patients felt that continued use of a DT and self-care routines are less important when they are well.

## Discussion

This study suggests that DT use in HF is not determined only by access, engagements or capability. Fear, trust, timing, and clinician recommendation shaped whether DTs were used and whether they supported self-care.

The findings are consistent with both HFA recommendations that HF education should be individualized, repeated, and embedded in self-care support,^3^ and with wider digital health literature showing that uptake is shaped by trust, perceived usefulness, digital literacy, and professional endorsement.^1,4^

The most actionable finding is that a DT recommendation may function as an information prescription. For patients, it may make a DT feel more reputable, help to reduce anxiety, and reduce some of the burden of having to judge the quality of the information for themselves. For clinicians, however, recommendation was limited by assumptions about capability, lack of consultation structure, and uncertainty about the evidence for DT use. This creates an inconsistency: patients with lower digital health literacy may have the most to gain from directed support,^8^ but may be least likely to receive it if clinicians assume they are unable or unwilling to use DT. Conversely, patients who appear digitally confident may still need guidance because confidence in using technology is not the same as competence in appraising complex information, especially when someone may have recently received a potentially life-changing diagnosis such as HF.

No patients or clinicians commented on HFmatters.org, the only DT that is recommended in international guidelines.^2^

Assumptions by clinicians were predominantly age-related and associated with the perceived inability or lack of desire to use DTs. Age bias in digital health has been reported before—age bias of clinicians was strongly predictive of negative clinician attitudes towards older adults using DTs in a recent European study.^9^ This age bias is likely a significant contributor to why so few patients aged older than 65 years (7.7%) were recommended a healthcare app compared with 45.3% in the 18–24 year age group in a UK-wide questionnaire study.^10^

The data supports simple service-level changes. Clinicians should ask simple questions about digital capability and preferences, such as how patients search for information and how they decide whether to trust it. HF services should agree on a small set of recommended DTs and include DT recommendations in early post-diagnosis education, when patients may be most open to new self-care routines. This may make DT prescriptions more consistent and equitable.

### Limitations

This was a mixed-methods study in three HF services in London, UK, at one point in time and may not generalize to all settings. Although the sample size was small, as is the best practice for qualitative studies, interviews continued until no new themes emerged and sampling deliberately captured variations in age, ethnicity, digital HL, and activation.

## Conclusion

Many patients living with HF express anxiety about the value of information provided by DTs with many withdrawing from accessing them. Clinician recommendations may help but require training, local consensus, and clear guidance from international bodies. Routine assessment of digital capability may address the inappropriate assumptions that clinicians appear to make about patients’ digital capability and their desire to use DTs, especially in older patients.

## Lead author biography


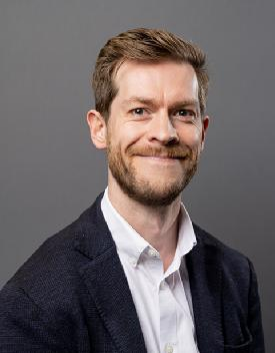


Dr Kyle McBeath is a cardiology consultant in London, UK, with sub-specialism in heart failure, advanced echocardiography, and valvular heart disease. He is a committee member of the British Society for Heart Failure and is passionate about the equitable digital transformation of cardiology care.

## Data Availability

The original recorded interview data underlying this article cannot be shared publicly due to the privacy of participants in the study. The coding framework will be shared upon reasonable request to the corresponding author.
